# The versatile roles of testrapanins in cancer from intracellular signaling to cell–cell communication: cell membrane proteins without ligands

**DOI:** 10.1186/s13578-023-00995-8

**Published:** 2023-03-20

**Authors:** Zhihang Zhou, Zihan Yang, Li Zhou, Mengsu Yang, Song He

**Affiliations:** 1 Department of Gastroenterology, The Second Affiliated Hospital of Chongqing Medical University, Chongqing, China; 2 Department of Biomedical Sciences, and Tung Biomedical Sciences Center, City University of Hong Kong, 83 Tat Chee Avenue, Kowloon, Hong Kong, SAR People’s Republic of China; 3 Department of Precision Diagnostic and Therapeutic Technology, City University of Hong Kong Futian Research Institute, Shenzhen, Guangdong China

**Keywords:** Tetraspanin, Cancer, Metastasis, Extracellular vesicles, Immunonology

## Abstract

The tetraspanins (TSPANs) are a family of four-transmembrane proteins with 33 members in mammals. They are variably expressed on the cell surface, various intracellular organelles and vesicles in nearly all cell types. Different from the majority of cell membrane proteins, TSPANs do not have natural ligands. TSPANs typically organize laterally with other membrane proteins to form tetraspanin-enriched microdomains (TEMs) to influence cell adhesion, migration, invasion, survival and induce downstream signaling. Emerging evidence shows that TSPANs can regulate not only cancer cell growth, metastasis, stemness, drug resistance, but also biogenesis of extracellular vesicles (exosomes and migrasomes), and immunomicroenvironment. This review summarizes recent studies that have shown the versatile function of TSPANs in cancer development and progression, or the molecular mechanism of TSPANs. These findings support the potential of TSPANs as novel therapeutic targets against cancer.

## Introduction

The tetraspanins (TSPANs) are a family of proteins with four transmembrane domains (TM1, TM2, TM3, and TM4), two extracellular loops (ECL1 and ECL2), and one intracellular loop (ICL) [1]. In Homo sapiens, the TSPANs family has 33 members, namely TSPAN1-TSPAN33 (Table 1). Some members have their common-used names, such as CD9 for TSPAN29, CD151 for TSPAN24, and CD81 for TSPAN28 (Table 1). 65 to 95% of amino acids are highly conserved among the TSPAN family members. The four transmembrane domains form a compact bundle in the membrane and facilitate interactions with other proteins [2]. TM domains contain many polar residues that can stabilize TSPAN protein structure with the help of ECL2 disulfide crosslinks. ECL2 is essential to the functions of TSPANs since most of protein–protein interaction sites have been mapped to ECL2. ECL2 consists of a conserved domain and a variable domain. The conserved domain facilitates interactions between different TSPAN molecules, while the variable domain determines interactions with other non-TSPAN proteins. There are also some highly conserved motifs in ECL2, such as CCG (Cys-Cys-Gly), PXSC (Phe-X-Ser-Cys) and EGC (Glu-Gly-Cys) [3]. These conserved motifs are basic structures for the interaction with other proteins. However, the structure and function of ECL1 and ICL have remained unclear so far [4]. The crystal structure of TSPAN proteins remains unknown until Rie Umeda and colleagues recently revealed the crystal structure of CD9 (TSPAN29) [5]. They found that the reversed cone-like molecular shape of CD9 in the crystalline lipid layers, giving reasons to the CD9 localization in regions with high membrane curvature and its implications in membrane remodeling [5].

**Table 1:** The TSPAN family members

Common name	TSPAN member	Alternative names	Gene ID	Uniprot code
TSPAN1	TSPAN1	TSP-1, NET-1, TM4-C, C4.8	10103	O60635
TSPAN2	TSPAN2	TSP-2, NET-3	10100	O60636
TSPAN3	TSPAN3	TSP-3, TM4-A, TM4SF8	10099	O60637
TSPAN4	TSPAN4	TSP-4, NAG-2, TM4SF7	7106	O14817
TSPAN5	TSPAN5	TSP-5, NET-4, TM4SF9	10098	P62079
TSPAN6	TSPAN6	TSP-6, TM4SF6, T245	7105	O43657
TSPAN7	TSPAN7	CD231,TALLA-1,A15, DXS1692E, MXS1, MRX58, TM4SF2, XLID58	7102	P41732
TSPAN8	TSPAN8	CO-029, TM4SF3	7103	P19075
TSPAN9	TSPAN9	NET-5, PP1057	10867	O75954
TSPAN10	TSPAN10	Oculospanin/OCSP	83882	Q9H1Z9
TSPAN11	TSPAN11	VSSW1971	441631	A1L157
TSPAN12	TSPAN12	NET-2, TM4SF12, EVR5	23554	O95859
TSPAN13	TSPAN13	NET-6, TM4SF13	27075	O95857
TSPAN14	TSPAN14	TM4SF14, MGC11352, DC-TM4F2	81619	Q8NG11
TSPAN15	TSPAN15	NET-7, TM4SF15	23555	O95858
TSPAN16	TSPAN16	TM4-B, TM4SF16	26526	Q9UKR8
TSPAN17	TSPAN17	FBXO23, TM4SF17	26262	Q96FV3
TSPAN18	TSPAN18	–	90139	Q96SJ8
TSPAN19	TSPAN19	–	144448	P0C672
TSPAN20	TSPAN20	UPK1B	7348	O75841
TSPAN21	TSPAN21	UPK1A	11045	O00322
TSPAN22	TSPAN22	RDS, PRPH2, CACD2, Rd2, RP7, AOFMD, MDBS1, AVMD, DS	5961	P23942
TSPAN23	TSPAN23	ROM1, ROSP1	6094	Q03395
CD151	TSPAN24	PETA-3, RAPH, SFA-1, GP27, EBS7, MER2	977	P48509
CD53	TSPAN25	MOX44	963	P19397
CD37	TSPAN26	GP52-40	951	P11049
CD82	TSPAN27	KAI1, SAR2, ST6, IA4, GR15	3732	P27701
CD81	TSPAN28	TAPA1, CVID6,S5.7	975	P60033
CD9	TSPAN29	MIC3, GIG2, P24, BTCC-1, DRAP-27	928	P21926
CD63	TSPAN30	MLA1,ME491, LAMP-3, OMA81H, LIMP1	967	P08962
TSPAN31	TSPAN31	SAS	6302	Q12999
TSPAN32	TSPAN32	TSSC6, PHEMX, CDNA 6, ART1, PHMX	10077	Q96QS1
TSPAN33	TSPAN33	PEN	340348	Q86UF1

**Fig. 1:**
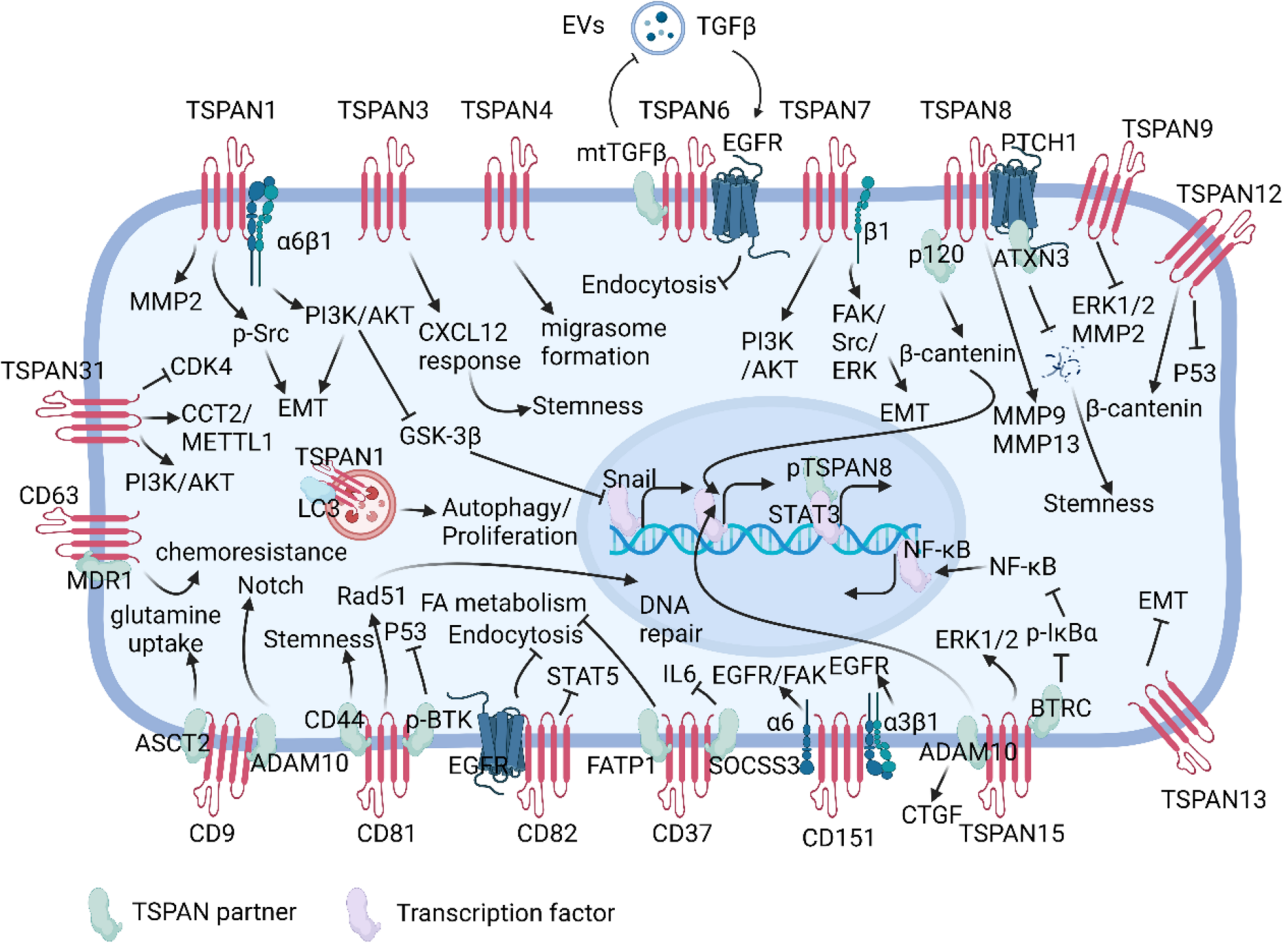
The intracellular signaling of TSPANs in cancer. Although mainly located on cell membrane, TSPANs have no natural ligands. They affect different biological processes mainly via interacting with different partner molecules to form TEMs. Integrins are the most prominent partner of TSPANs. Other partners of TSPANs include growth factor receptors (EGFR [9], mtTGF-β [10]), transporters (ASCT2 [11], FATP1 [12], MDR1 [13]), membrane-linked kinases (BTRC [14], SOCSS3 [15], ATXN3 [16]), other transmembrane proteins (ADAM10[17], CD44 [18], p120 [19]). Thus, TSPANs can affect several signaling pathways, including PI3K/AKT, Wnt/β-catenin, ERK1/2, STAT3/5, Src, Notch pathways

TSPANs are expressed on the surface of most nucleated cells and play important roles in cell proliferation, differentiation, adhesion, migration, and cell–cell crosstalk [6]. Recent studies have revealed that TSPANs are indispensable for cancer initiation and progression [3]. These members have pro-tumor or anti-tumor functions in a context-dependent manner [3]. Although mainly located on cell membrane, TSPANs have no natural ligands. They affect different biological processes mainly via interacting with different partner molecules to form tetraspanin-enriched microdomains (TEMs). Tetraspanins can further influence the distribution and function of their partners. Integrins are the most prominent partner of TSPANs [3]. For instance, CD151 (TSPAN24) can enhance integrin-mediated adhesion to laminin and downstream signaling [7]. CD151 can form a complex with integrin α3β1 to activate PI3K or PI4K signaling pathway, and finally impacts cancer cell migration via remodeling actin cytoskeleton or inducing matrix metalloproteinase (MMP) secretion. Furthermore, CD151–α3β1/α6β4 integrin complexes can recruit and activate small G proteins (RAS, RAC1, and CDC42) in melanoma cell lines [8]. Other partners of TSPANs include growth factor receptors (EGFR [9], mtTGF-β [10]), transporters (ASCT2 [11], FATP1 [12], MDR1 [13]), membrane-linked kinases (BTRC [14], SOCSS3 [15], ATXN3 [16]), other transmembrane proteins (ADAM10 [17], CD44 [18], p120 [19]) or some nonproteins such as cholesterol [20]. For example, TSPAN6 could bind with EGFR and inhibit its downstream KRAS-ERK1/2 signaling to suppress KRAS-driven cancer initiation and metastasis [20](Fig. 1).

Moreover, the post-translational modifications at specific sites in TSPAN proteins are important for the protein–protein interaction and the subsequent downstream pathways. These modifications include N-glycosylations at the ECL2 domain, palmitoylation at the N- and C-terminal tails and ubiquitination at the N-terminal domain [3]. For example, glycosylation of CD63 (TSPAN30) in breast cancer cells by RPN2, part of the N-oligosaccharyle transferase complex, could stabilize CD63 on the cell membrane [13]. Glycosylation of TSPAN1 promotes its transition through the endoplasmic reticulum in ovarian cancer cells [21], while glycosylation of CD82 by the glycosyltransferase MGAT3 is pivotal to disrupt integrin α5β1-mediated cell migration [22].

The multiple possible combinations between different TSPANs and their interacting proteins could generate an enormous variability of biological function. In addition to the traditional role of TSPANs in cancer growth, invasion and metastasis, recent studies have revealed that these proteins also participate in cancer stemness, cell–cell communication, drug resistance and cancer immunology [16, 23–26] (Table 2). Specifically, TSPANs have been shown to regulate the biogenesis, cell-specific attachment of exosomes[19], and TSPAN4 can even promote the formation of migrasomes [27]. This review summarizes recent studies that have shown the versatile role of tetraspanins in cancer biology.

**Table 2:** The function and regulatory mechanism of TSPAN members in cancer

Common name	Family name	Refs.	Year	Cancer Type	Function	Protein partner	Downstream signaling	Upstream regulation
TSPAN1	TSPAN1	[36]	2009	OVC	Elevated expression in advanced stage ovarian cancer	–	–	–
		[38]	2010	CRC	Promotes proliferation and invasion	–	–	–
		[35]	2015	GC	Promotes proliferation and invasion	–	–	miR-573
		[39]	2017	PC	Promote migration and EMT	–	–	androgen
		[33]	2018	CCA	Promote growth, EMT and metastasis	α6β1	PI3K/AKT	–
		[116]	2018	CRC	Upregulated in EVs	–	–	–
		[34]	2019	PDAC	Promote migration and invasion	–	MMP2	–
		[93]	2020	HNSCC	Promote resistance to cisplatin, proliferation, inhibit apoptosis	–	EMT SRC signaling	–
		[79]	2021	PDAC	Promote proliferation	LC3	Autophagy	miR454
		[37]	2021	BC	Promote proliferation, migration and EMT	–	PI3K/AKT	–
TSPAN3	TSPAN3	[91]	2015	AML	Enhance stemness	–	CXCL12	Musashi 2
		[101]	2020	AML	Promote adriamycin resistance, proliferation, migration and invasion and reduced apoptosis	–	–	miR-193a-3p
TSPAN4	TSPAN4	[27]	2019	BC	Promote migrasome formation	–	increase membrane stiffness	–
		[118]	2022	GC	Promote membrane repair	–	–	–
TSPAN6	TSPAN6	[83]	2021	CRC	Inhibit carcinogenesis	tmTGFa	EGFR	–
		[20]	2022	KRAS driven cancer	Inhhibit tumor growth and metastasis	EGFR	RAS	–
TSPAN7	TSPAN7	[74]	2015	Myeloma	Promote cell adhesion to stromal cells, transendothelial migration, and in vivo metastasis, not affect proliferation	–	–	–
		[73]	2018	NSCLC	Promote proliferation, migration, and EMT process	–	–	–
		[75]	2020	Bladder Cancer	Inhibit growth and invasion	–	PI3K/AKT	–
		[72]	2022	Osteosarcoma	Promote migration, invasion, EMT and metastasis	β1 integrin	FAK/SRC/RAS/ERK	–
TSPAN8	TSPAN8	[45]	2013	Rat PDAC	Promote metastasis	–	MMP9/13	–
		[16]	2019	BC	Promote stemness and drug resistance	PTCH1	Heghog signaling	–
		[19]	2019	BC	Promote MET, cell–cell adhesion and EV production; inhibit motility	p120	β-cantenin	–
		[43]	2019	Melanoma	Promote invasion	–	β-cantenin	b-cantenin
		[41]	2020	CRC	Promote proliferation, migration and EMT process	–	–	LSD1
		[103]	2020	PDAC	Promote endothelial cell maturation via EVs	–	–	–
		[42]	2021	PDAC	Promote metastasis	–	–	SOX2
		[44]	2021	Melanoma	Correlate with high metastatic risk and poor prognosis	–	–	–
		[9]	2022	BC	Nuclear localization promotes invasion and metastasis	STAT3	STAT3 signaling	EGFR
TSPAN9	TSPAN9	[70]	2016	GC	Inhibit proliferation and migration	–	ERK1/2 MMP2	–
TSPAN12	TSPAN12	[49]	2014	BC	Inhibit tumor growth, while promote metastasis	–	canonical Wnt-pathway signaling	–
		[76]	2017	SCLC	Promote chemoresistance, proliferation and tumor growth	–	–	miR-495
		[48]	2017	CRC	Promote proliferation, migration and invasion, in vivo tumor growth, while reduce cell apoptosis	–	–	–
		[50]	2018	NSCLC	Promote proliferation, and in vivo tumor growth, while increase apoptosis	–	p53	–
		[77]	2020	NSCLC	Inhibit tumor growth	–	–	miR-196b-5p
TSPAN13	TSPAN13	[66]	2018	Sarcoma	Promote invasion, and inhibit apoptosis	–	EMT	h-TERT
TSPAN15	TSPAN15	[14]	2018	ESCC	Promote metastasis	BTRC	NF-kB	miR-339-5p
		[17]	2019	HCC	Promote proliferation	ADAM10	ERK1/2 and CTGF secretion	–
		[56]	2019	ESCC	Promote invasion and migration, did not affect proliferation	–	increase ADAM10 at cell surface, and β-catenin activation	–
CD151	TSPAN24	[53]	2008	BC	Promote migration and invasion	α6	EGFR/FAK	–
		[45]	2013	Rat PDAC	Promote metastasis	–	integrin	–
		[55]	2021	NSCLC	Promote migration and invasion	α3β1	EGFR	–
		[111]	2021	TNBC	Increased in EVs	–	–	–
		[80]	2022	Osteosarcoma	Promote tumor growth	–	c-Myc/ SPTCL1 /sphingolipid synthesis	–
CD37	TSPAN26	[85]	2016	BCL	Predicts favorable prognosis	–	–	–
		[15]	2016	BCL	Inhibit tumor development	SOCSS3	IL-6 signaling	–
		[12]	2022	BCL	Inhibit proliferation	FATP1	inhibit FA metabolism	–
CD82	TSPAN27	[67]	2000	NSCLC	Inhibit metastasis	EGFR	EGFR endosytosis	–
		[69]	2003	TCL/PC	Inhibit migration	EWI2	–	–
		[92]	2014	AML	Enhance stemness	–	STAT5/IL10	–
		[102]	2020	AML	Promote daunorubicin resistance	–	PKC/integrin b/p38	–
		[22]	2020	OVC	Inhibit migration and etastasis	–	–	glycosylation by MGAT3
		[68]	2021	Breast epithelia	Increase adhesion and lamapodia, and inhibit migration	–	YAP	–
CD81	TSPAN28	[99]	2020	ALL	Enhance chemoresistance	–	BTK	–
		[58]	2021	TNBC	Promote metastasis	–	–	–
		[100]	2021	GBM	Ehance radioresistance	–	Rad51 translocation	–
		[18]	2022	TNBC	Promote stemness and metastasis	CD44	EV integrity	–
CD9	TSPAN29	[59]	2012	BC	Overexpressed in bone metastases	–	–	–
		[61]	2014	BC	Promote mitosis; Nuclear localization	–	–	–
		[11]	2019	PDAC	Enhance stemness	ASCT2	glutamine uptake	–
		[60]	2020	BC	Promote migration and in vivo tumor growth, but not tumor initiation or metastasis	–	–	miR-518f-5p
		[90 ]	2021	AML	Promote chemoresistance and stemness	–	–	–
		[81]	2022	CRC	-	ADAM10	Notch signaling	–
CD63	TSPAN30	[13]	2014	BC	Promote docetaxel resistance	MDR1	–	glycosylation
		[71]	2014	Melanoma	Inhibit cell motility, invasion and in vivo tumor growth	–	–	–
TSPAN31	TSPAN31	[65]	2017	HCC	Promote migration, but not affect proliferation	–	PI3K/AKT	miR-135b
		[82]	2020	Cervical cancer	Inhibit proliferation	–	natural antisense transcript to inhibit CDK4 expression	–
		[63]	2022	GC	Promote proliferation and migration	–	METTL1/CCT2	–
		[64]	2022	GC	Promote proliferation, migration and chemoresistance	–	PI3K/AKT ABCC2	–

*BC* breast cancer, *GC* gastric cancer, *PDAC* pancreatic ductal carcinoma, *OVC* ovarian carcinoma, *TNBC* triple-negative carcinoma, *ESCC* esophagus squamous cell carcinoma, *NSCLC* non-small cell lung cancer, S*CLC* small cell lung cancer, AML acute myeloid leukemia, *BCL* B cell lymphoma, *TCL* T cell leukemia, HNSCC head and neck squamous cell carcinoma, *CCA* cholangiocarcinoma, *PC* prostate cancer, *FA* fatty acid

### TSPANs in cancer invasion and metastasis

Metastasis is the leading cause of cancer-related deaths and the failure of cancer treatment [28]. The development of metastases requires cancer cells to leave their primary site (local invasion), intravasate, circulate and survival in the bloodstream, extravasate, acclimate in a secondary site, and finally form new colonization foci [29, 30]. Indeed, all cellular behaviors, including cancer cell migration, invasion, proliferation, apoptosis, should be concisely coordinated to ensure cancer metastasis [31, 32]. The function of TSPAN members in cancer cell invasion and metastasis will be first worth summarizing.

Most TSPAN proteins are reported to promote cancer invasion and metastasis, such as TSPAN1, TSPAN8, TSPAN12, TSPAN15, CD151, CD81, CD9, TSPAN31, and TSPAN13. TSPAN1 is mainly expressed on the plasma membrane, some intracellular vesicles and organelles (e.g., exosomes, lysosomes and endoplasmic reticulum), and perinuclear membrane [3]. TSPAN1 is frequently upregulated in cholangiocarcinoma, pancreatic cancer, and gastric cancer [33–35]. High level of TSPAN1 correlates with advanced tumor stage and metastasis [33, 36]. TSPAN1 could promote cholangiocarcinoma growth, metastasis, and induce epithelial-to-mesenchymal transition (EMT) by interacting with integrin α6β1 to amplify the PI3K/AKT/GSK-3β/Snail/PTEN feedback loop [33]. Meanwhile, TSPAN1 promotes pancreatic cancer cell migration and invasion by upregulating MMP2 [34]. The ablation of TSPAN1 suppressed the growth and motility of breast cancer cells by inhibiting the EMT process and the PI3K/Akt pathway [37]. TSPAN1 also significantly promotes the proliferation and invasion of colon cancer cells [38] and gastric cancer cells [35] with unrevealed mechanism. Jennifer and colleagues also revealed that TSPAN1 could promote the migration of prostate cancer cells [39]. Furthermore, TSPAN-1 was found to interact with and stabilize the human thiamine transporter-1 (hTHTR-1) to facilitate thiamine intake in colon cancer and epithelial cells [40].

TSPAN8 is reported to be highly expressed in colorectal cancer, pancreatic cancer tissues and melanoma [41–44]. TSPAN8 promotes the proliferation, migration and EMT process of colorectal cancer cells [41]. TSPAN8 also facilitates metastasis of pancreatic cancer cells in vivo and in vitro [42], while it promotes metastasis of rat pancreatic cancer cells through recruiting integrins out of adhesion into motility promoting complexes [45]. Meanwhile, TSPAN8 could induce the EMT process and enhance cell–cell adhesion of breast cancer cells via interacting with p120 [19]. TSPAN8 is more frequently expressed in metastatic melanoma species and correlates with the presence of a BRAF-V600E mutation, a higher propensity to form distant metastases and an increased risk of death [44]. TSPAN8 stabilizes β-catenin, which in turn directly triggers the transcription of TSPAN8, leading to melanoma invasion [43]. TSPAN8^+^ melanoma cells have elevated active MMP-3 and low TIMP-1 levels to promote keratinocyte-originated proMMP-9 activation process, collagen IV degradation and dermal colonization [46]. Interestingly, the nuclear localization of TSPAN8 can be detected in multiple cancer cells, which involves the formation of TSPAN8-cholesterol-14-3-3θ-importin β complex after being palmitoylated [47]. The same group further demonstrated that nuclear TSPAN8 could interact with STAT3 to enhance its chromatin occupancy [9]. The authors further revealed that blocking the translocation of TSPAN8 using a humanized monoclonal antibody hT8Ab4 can remarkably inhibit breast cancer growth in vitro and in vivo [9].

TSPAN12 is highly expressed in both colorectal cancer and non-small lung cancer tissues [48–50]. High TSPAN12 expression is significantly correlated with TNM stage, tumor size and lymph node metastasis in colorectal cancer patients. Knockdown of TSPAN12 significantly could suppress cell proliferation, migration and invasion, in vivo tumor growth, while induce cell apoptosis of both colorectal cancer and non-small cell llung cancer cells [48, 50]. In contrast, TSPAN12 promotes breast cancer cell growth, but depresses tumor-endothelial interactions and metastasis to mouse lungs. Mechanistic study demonstrated that TSPAN12 stabilizes FZD4–LRP5 association to activate the canonical Wnt-pathway signaling [49].

CD151 expression has been reported to be associated with advanced cancer stage, cancer invasiveness and poor prognosis in endometrial cancer, hepatocellular carcinoma, breast cancer and non-small cell lung cancer patients [45, 51–55]. CD151 ablation markedly reduces breast cancer cell migration, invasion by inhibiting FAK-Rac1 signaling and disrupting EGFR-α6 integrin collaboration [53]. Meanwhile, CD151 could promote non-small cell lung cancer cell proliferation, migration, and invasion by interacting with integrin α3β1 to enhance EGFR signaling [55]. Moreover, CD151 recruits and activates MMP9 and MMP13 to create a path for invasion and metastasis of rat pancreatic cancer cells [45].

High TSPAN15 expression in esophageal squamous cell carcinoma tissues is significantly associated with lymph node and distant metastasis, and poor prognosis [14, 56]. TSPAN15 could augment metastatic capabilities but not proliferation of esophageal squamous cell carcinoma cells. It specifically interacts with β-transducin repeat containing E3 ubiquitin protein ligase (BTRC) to promote the ubiquitination and proteasomal degradation of p-IκBα, and thereby triggers NF-κB nuclear translocation and initiates transcription of several metastasis-related genes [14]. TSPAN15 can also increase the ADAM10 on the cell surface, the soluble N-Cadherin secretion and β-catenin nuclear translocation in esophagus cancer cells [57].

CD81, CD9, TSPAN31, and TSPAN13 are also reported to facilitate cancer cell invasion and metastasis. Mice with C81 deficient develop fewer breast cancer metastases compared to their wild-type counterparts. The same group showed that a unique anti-human CD81 antibody (5A6) effectively halts invasion and metastasis of triple-negative breast cancer cell lines [58]. CD9 is highly expressed in the bone metastases versus primary breast cancer tissues [59]. It is reported to promote breast cancer migration [60]. However, CD9 deletion in the MMTV/PyMT mouse model impaired tumor growth, but did not affect tumor initiation or metastasis [60]. CD9 depletion or anti-CD9 antibody could result in polynucleation and multipolar mitoses [61]. CD9 on lung adenocarcinoma cells is also necessary for the pro-invasion effect of the secreted TIMP-1 from cancer-associated fibroblasts, probably depending on the direct interaction between these two proteins [62]. TSPAN31 is highly expressed in gastric cancer tissues and correlates with poor prognosis of gastric cancer patients. It could promote the gastric cancer proliferation and migration via activating PI3K/AKT signaling [63, 64]. In contrast, TSPAN31 facilitates the migration and invasion, but has less impact on proliferation of hepatocellular carcinoma cells [65]. Furthermore, knockdown of TSAPN13 in U2OS sarcoma cells increased cell apoptosis and also suppressed EMT process [66].

On the other hand, some TSPAN family members, such as CD82, TSPAN6, TSPAN9 and CD63, exhibit tumor suppressor properties. For example, CD82 directly associates with EGFR and suppressed EGF-induced lamellipodial extensions and cell migration in non-small cell lung cancer cells. CD82 could specifically increase EGFR endocytosis after EGF stimulation but not the initial activation of EGFR [67]. It also inhibits cell migration by enhancing focal adhesion through promoting YAP nuclear translocation in breast epithelial cells [68]. In addition, CD82 binds with EWI2 in Du145 metastatic prostate cancer cells and inhibits cell migration on both fibronectin- and laminin-coated substratum [69]. The other member TSPAN6 could suppress tumor growth and metastasis of human RAS activating mutant pancreatic cancer xenografts. Whole-body knockout as well as tumor cell autonomous inactivation using floxed alleles of TSPAN6 in mice enhanced Kras^G12D^-driven lung tumor initiation and malignant progression. Similar to the function in lung cancer cells, TSPAN6 binds to the EGFR and blocks EGFR-induced RAS activation, thus inhibiting EMT process and cell migration [20]. The proliferation, migration and invasion of human gastric cancer SGC7901 cells were significantly inhibited by overexpression of TSPAN9, which is mediated by the inhibition of ERK1/2 signaling and MMP-9 expression [70]. Finally, CD63-silenced melanoma cells showed enhanced motility, invasiveness, EMT and in vivo tumor growth [71].

Evidence has shown the context-dependent role of TSPAN7 in cancer. TSPAN7 is highly expressed in primary osteosarcomas and promote osteosarcoma cell growth, EMT process, and in vivo metastasis. Mechanistically, the authors demonstrated that TSPAN7 interacted with β1 integrin to activate FAK-Src-Ras-ERK1/2 signaling [72]. TSPAN7 could also promote non-small cell lung cancer cell proliferation, migration, and EMT process [73]. However, the tumor-suppressing effect of TSPAN7 has been reported in myeloma and bladder cancer. TSPAN7 significantly reduced tumor burden in 5TGM1/KaLwRij mice 4 weeks after intravenous injection of the murine myeloma cell line 5TGM1 by increasing cell adhesion to stromal cells and transendothelial migration, with no impact on cell proliferation [74]. Additionally, Xi Yu et al. showed low TSPAN7 expression level is associated with higher tumor stage and poor prognosis in bladder cancer and TSPAN7 inhibits both cell migration and proliferation through suppressing the PTEN/PI3K/AKT Pathway [75].

In summary, most TSPAN proteins (TSPAN1, TSPAN8, TSPAN12, TSPAN15, CD151, CD81, CD9, TSPAN31, TSPAN13) could promote cancer invasion and metastasis, while few members (CD82, TSPAN6, TSPAN9 and CD63) have the opposite function. Meanwhile, the function of TSPAN7 in cancer invasion and metastasis is context-dependent.

### TSPANs in cancer proliferation and growth

As mentioned above, several TSPAN family members could regulate the invasion and metastasis of cancer cells. Cell growth and proliferation are also essential for cancer metastasis or progression besides invasion. These biological processes should coordinate with each other to achieve cancer progression. We can see that TSPAN1, TSPAN12, TSPAN13, TSPAN6, TSPAN8, TSPAN9, CD151, CD63 can promote or inhibit both cancer cell invasion and proliferation. However, the function of TSPAN molecules seems to be context-dependent. For example, TSPAN31 has been reported to promote migration of hepatocellular carcinoma, but not affect cell proliferation [65]. Even more, TSPAN12 could inhibit growth of breast cancer cells, but enhance metastasis [49]. In contrast, TSPAN12 has been found to promote the proliferation of small cell lung cancer cells and colorectal cancer cells [48, 76]. Larger discrepancy exists as Hu Z et al. reported that TSPAN12 could promote the proliferation of non-small lung cancer cells [50], while another group found an opposite effect [77]. We herein summarize several studies that have focused on the function of TSPAN members in cancer cell proliferation and growth.

On the one hand, some TSPAN members could promote cancer cell growth. Pancreatic cancer is one of the most aggressive malignancy, with a 5-year survival rate of less than 5% [78]. High TSPAN1 expression was correlated with poor overall survival of pancreatic cancer patients, and TSPAN1 promote the proliferation of pancreatic cancer cells [79]. The authors further revealed that TSPAN1 promoted autophagy maturation via direct binding to LC3 by two conserved LC3-interacting regions in the two extracellular loops [79]. Moreover, TSPAN15 has been reported to promote the proliferation of hepatocellular carcinoma cells via activating ERK1/2 signaling [17]. However, it does not significantly affect the proliferation of esophagus carcinoma cells [55]. It can associate with a molecular scissor, ADAM10, but the effect is unknown [17, 57]. A latest study reported that CD151 could stabilize the oncogene c-Myc to activate the transcription of SPTLC1, the first rate-limiting enzyme in sphingolipid biosynthesis, thus fueling osteosarcoma cell growth [80]. Another recent study reported that TSPAN29 associates with ADAM10 to increase its cell surface trafficking and α-secretase activity, which further produces more cleaved Notch1 to support growth of colorectal cancer cells [81].

On the other hand, some other TSPAN members could inhibit cancer cell growth. TSPAN31 serves as a natural antisense transcript to inhibit CDK4 protein expression in human cervical cancer and hepatocellular carcinoma by targeting the 3ʹ-untranslated region of the CDK4 mRNA, thus suppressing cell proliferation [65, 82]. The expression of TSPAN6 is frequently decreased or even lost in colorectal cancer tissues, and correlates with favorable survival. TSPAN6 deletion facilitates colorectal cancer development and results in the activation of EGF-dependent signaling pathways through increased production of the transmembrane form of TGF-α (tmTGF-α) associated with extracellular vesicles [83]. Liang G. and colleagues found that TSPAN12 could inhibit tumor growth of non-small lung cancer cells [77]. CD9 associates with transmembrane TGF-α to enhance the ligand-induced activation of the EGFR, and thus promoted Madin-Darby Canine Kidney cells (MDCK) cell proliferation [84]. CD37, whose expression correlates with favorable prognosis, can protect against the development of B cell lymphoma by interacting with the suppressor of cytokine signaling 3 (SOCS3) to inhibit IL-6 signaling [15, 85]. Most recently, CD37 has been demonstrated to inhibit fatty acid metabolism in aggressive B-cell lymphoma through interacting with fatty acid transporter protein 1 (FATP1) in the plasma membrane, and inhibiting the uptake and processing of exogenous palmitate [12].

Altogether, TSPAN1, TSPAN15, TSPAN29, and CD151 could support cancer cell growth, while TSPAN31, TSPAN6, CD9, CD37 could inhibit cancer growth.

### TSPANs in cancer cell stemness

Stem cells are capable of both self-renewing and multilineage differentiating. Tumor heterogeneity is now recognized as a hallmark of tumors [86]. Only a distinct population of cancer cells has the capabilities of self-renewal, drug-resistance, metastasis and tumorigenicity, called cancer stem cells (CSCs) [87–89]. Our understanding of the biology and therapeutic implication of CSCs is still evolving since the establishment of this concept.

Several TSPAN members, such as CD9, TSPAN8, TSPAN3, CD82, CD81, and TSPAN1, have been implicated in the regulation of CSCs. CD9 has been reported to be specifically expressed on leukemia stem cells. CD9^positive^ cells exhibit more resistance to chemotherapy drugs, higher migration potential, and stronger tumorigenicity [90]. CD9 was also identified as a marker of pancreatic cancer-initiating cells. CD9^high^ pancreatic cancer cells have increased organoid formation capability and in vivo carcinogenesis. Mechanistically, CD9 enhances glutamine uptake in pancreatic cancer cells via promoting the plasma membrane localization of the glutamine transporter ASCT2 [11]. TSPAN8 expression is upregulated in breast CSCs. It could upregulate the stemness gene NANOG, OCT4, and ALDHA1, and enhance both tumor formation and drug resistance. TSPAN8 interacts with the Hedgehog receptor PTCH1 and inhibits the degradation of the SHH/PTCH1 complex through recruitment of deubiquitinating enzyme ATXN3, thus inducing downstream gene expression [16]. In addition, TSPAN3 knockout impaired leukemia stem cell self-renewal and disease propagation, and significantly improved survival in mouse models of acute myelocytic leukemia. This effect is at least partially mediated by disabling homing within in the niche in responses to CXCL12 [91]. CD82 was up-regulated in CD34^+^/CD38^+^ acute myelocytic leukemia stem cells and increased the phosphorylation of transcription factor STAT5 to transactivate IL-10 transcription [92]. More recently, CD81 has been revealed to interact with CD44 to enhance the stemness of triple-negative breast cancer cells, and high CD81 expression can be found in circulating tumor cells [18]. Furthermore, TSPAN1 is found to be elevated in CSCs from head and neck squamous cell carcinoma cells and lead to drug resistance [93].

CD63 and CD81 have also been reported to play important roles in stemness maintenance of non-malignant cells. CD63 could confer hematopoietic stem cells with more quiescent status, more robust self-renewal and myeloid differentiation abilities than those with negative/low CD63 expression. Knockout of CD63 in mice reduced the number of hematopoietic stem cells in bone marrow and CD63-deficient hematopoietic stem cells exhibit impaired quiescence and long-term repopulating capacity, and increased sensitivity to irradiation or 5-fluorouracil treatment. CD63 was found to interact with TGF-β receptors I and II to sustain TGF-β signaling activity [70]. A CD81^+^/PDGFRA^low^ population present just below crypts is sufficient to expand intestinal stem cells in vitro and contribute to stemness maintenance in vivo via secreting the BMP antagonist Gremlin1 [94].

### TSPANs in therapy resistance

Despite significant advances in cancer treatment, the development of resistance almost invariably emerges [95]. Multiple studies have revealed that cancer cells utilize a plethora of distinct mechanisms to survive under chemotherapy or radiotherapy [96, 97]. The following TSPANs have been involved in enhancing cancer therapy resistance: CD9, CD81, TSPAN1, TSPAN3, TSPAN31, CD82 and CD63.

CD9 mediates chemoresistance in acute myeloid leukemia [90] and small cell lung cancer [98]. As mentioned above, CD9 enhances the stemness and chemoresistance of acute myeloid leukemia cells. In addition, CD9 is expressed preferentially in relapsed small cell lung cancers but not chemo-responsive primary tumors. CD9 renders small cell lung cancer cells resistant to cisplatin or etoposide, and increases cell adherence to fibronectin via β1 integrin. A specific monoclonal antibody against CD9, ALB6, triggered apoptosis in the chemoresistant cells [98]. CD81 has been reported to enhance both chemoresistance and radioresistance. CD81 knockout induces chemosensitivity, reduces cellular adhesion, and disrupts in vivo bone marrow homing and engraftment in acute lymphoblastic leukemia cells. This chemosensitization is mediated through control of Bruton tyrosine kinase (BTK) signaling and induction of p53-mediated cell death [99]. Accordingly, suppressing CD81 by siRNA/shRNA could enhance radiation-induced cell killing and DNA damage of γ-H2AX formation, and delaye tumor xenograft growth of glioblastoma. Knockdown of CD81 significantly decreased radiation-induced expression of nuclear Rad51, a key protein for homologous recombination repair [100].

TSPAN1, TSPAN3, TSPAN12, TSPAN31, CD82 and CD63 have also been reported to reduce chemosensitivity of cancer cells. TSPAN1 is found to be upregulated in cisplatin-resistant head and neck squamous cell carcinoma cells. TSPAN1 depletion reduces cell proliferation, induces apoptosis, decreases autophagy, sensitizes to chemotherapeutic agents and inhibits the phosphorylation of SRC signaling [93]. Moreover, TSPAN3 is up-regulated in adriamycin-resistant acute myeloid leukemia samples and cells. It increases adriamycin resistance, proliferation, migration and invasion and reduces apoptosis in adriamycin-resistant cells [101]. TSPAN12 elevation in small cell lung cancer specimens correlates with poor pathologic stage and shorter survival time. It could enhance cells chemoresistance, proliferation and tumor growth [76]. Knockdown of TSPAN31 improves chemosensitivity to cisplatin through the suppression of ABCC2 in gastric cancer cells [64]. Furthermore, CD63 silencing reduces the chemoresistance of breast cancer cells by stabilizing MDR1 on cell surface [13]. CD82 could significantly reduce cell death in response to daunorubicin in acute myeloid leukemia cells. The underlying mechanism involves the activation of protein kinase c alpha (PKCα)-β1 integrin-p38 signaling [102].

### TSPANs in extracellular vesicles

**Fig. 2:**
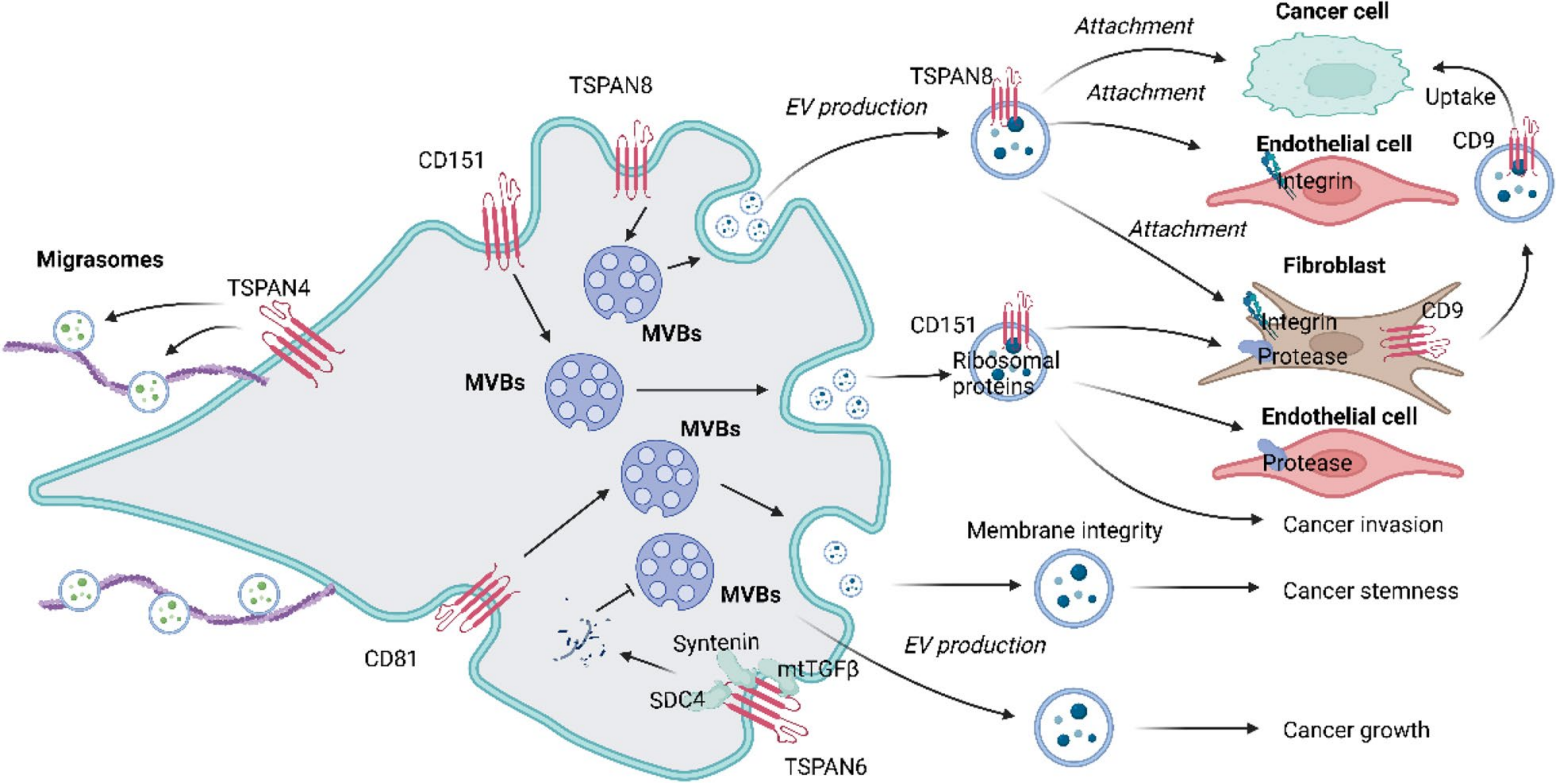
The function of TSPANs in EV biogenesis. TSPANs can regulate the biogenesis of EVs in the following aspects: (1) TEMs are prone for internalization or curvature [106, 107], and proteins in TEMs have been proposed to be carried by EVs [108]; (2) TSPANs contribute to EE traffic towards MVB [109]. (3) The exosome TSPAN web strengthens binding avidity by clustering TSPAN-associated molecules. TSPAN8 could promote EV production and the attachment to target cells. In contrast, TSPAN6 can reduce EV production and the contents in EVs in cancer cells. CD151 facilitates secretion of ribosomal proteins while reducing complement proteins to promote the migration and invasion of triple-negative breast cancer cells [111]. CD81 ensures the membrane integrity of exosomes, which are capable of inducing stemness in triple-negative breast cancer [18]. CD9 on the surface of EVs facilitates the uptake of EVs from cancer-associated fibroblasts by pancreatic cancer cells [117]

Extracellular vesicles (EV) are of utmost importance in intercellular communication under physiological and pathological conditions, allowing cells to exchange proteins, lipids, and genetic material [93]. Exosomes are a subpopulation of small 40–100 nm EVs, which can be recovered in all body fluids. Exosomes are build-up by a transmembrane protein-containing lipid bilayer and proteins, coding and noncoding RNA, and DNA in the vesicle lumen. Exosome biogenesis starts with early endosomes (EE) formation, which originate from the trans-Golgi network or internalized membrane microdomains [104]. EE are then guided towards multivesicular bodies (MVB) to receive their cargo during inward budding of intraluminal vesicles [105]. Several TSPANs such as CD9, CD81, and CD63 are major constituents and canonical markers of EVs. Moreover, they can regulate the biogenesis of exosomes in the following aspects: (1) TSPAN-enriched microdomains (TEMs) are prone for internalization or curvature [106, 107], and proteins in TEMs have been proposed to be carried by EVs [108]; (2) TSPANs contribute to EE traffic towards MVB [109]. (3) The exosome TSPAN web strengthens binding avidity by clustering TSPAN-associated molecules [110] (Fig. 2).

TSPAN8 has been reported to promote EV production and the attachment to target cells. TSPAN8 mediated a several-fold increase in EV number in breast cancer cell culture and the circulation of tumour-bearing animals [19]. Rat PDAC cells with CD151 or TSPAN8 knockdown poorly metastasize, but regain metastatic capacity when rats are pretreated with exosomes from parental cells. Both exosomal CD151 and TSPAN8 contribute to host matrix remodelling due to exosomal TSPAN-integrin and TSPAN-protease associations, and stroma cell activation [112]. Extracellular vesicles originated from TSPAN8^high^ expression pancreatic cancer cells remarkably promotes maturation, activation of endothelial cells and fibroblasts [103]. Tspan8-enriched EVs exhibit stronger attachment to the target cells including breast cancer cells or fibroblast, by molecular adhesion [25]. TSPAN8-overexpressing EVs increases invasion of non-small lung cancer cells and elevates TSPAN8 expression on serum EVs correlated with reduced distant metastasis-free survival [113].

In contrast, TSPAN6 can reduce EV production and the contents in EVs in cancer cells. TSPN6 acts as a suppressor of exosome release by facilitating the lysosomal degradation of SDC4 and syntenin in breast cancer cells [114]. In addition, TSPAN6 deletion promotes colorectal cancer organoid growth in an EV-dependent manner, as EV-depleted media could not support proliferation and viability of TSPAN6-expressing organoids [83]. Furthermore, TSPAN6 in colorectal cancer cells might decrease the TGF-α content in the EVs, which could activate EGFR signaling in target cells. However, TSPAN6 recruits the cytosolic exosome-forming adaptor syntenin to increase secretion of exosomes that contain amyloid precursor protein-C-terminal fragments in brain [115].

CD151, TSPAN1, CD81, CD9 have also been implicated in the EV biogenesis. CD151 expression level in triple-negative breast cancer-derived serum exosomes is significantly higher than those from healthy subjects [111]. CD151 facilitates secretion of ribosomal proteins while reducing complement proteins to promote the migration and invasion of triple-negative breast cancer cells [111]. TSPAN1 was found to be upregulated in plasma EVs from colon cancer patients compared to those from healthy controls [116]. In addition, CD81 has been reported to ensure the membrane integrity of exosomes, which are capable of inducing stemness in triple-negative breast cancer [18]. Recently, Jérémy Nigri and colleagues showed that CD9 on the surface of EVs facilitates the uptake of EVs from cancer-associated fibroblasts by pancreatic cancer cells [117].

TSPAN proteins also regulate the formation of migrasomes [27]. Migrasomes are large vesicle-like structures that are released from cells during migration, providing spatiotemporal chemical information for cell–cell communication. The authors found that 14 out of the 33 known TSPANs could enhance migrasome formation [27]. TSPAN4, one of the most powerful promoters of migrasome formation, could elevate the membrane stiffness of the TEMs to facilitate micron-scale macrodomain assembly [27]. The same group further reported that TSPAN4 promotes membrane repair by mediating assembly of micron-scale macrodomains in gastric cancer cell, rat kidney cell and mouse fibroblast [118].

### Tetraspanins in cancer immunology

Immunotherapy has revolutionized and rejuvenated cancer treatment. The immune system plays a pivotal role in immunosurveillance, as immune cells of the adaptive and innate immune systems infiltrate into the tumor microenvironment (TME) and modulate cancer progression. Due to their powerful tumor-killing capability, T cells are the focus of tumor immunology [119]. Antigen presentation is necessary for T cell immune surveillance of cancer cells. CD8 + T cell activation is primarily driven by the presentation of peptides from endogenously expressed proteins on MHC class I molecules (MHC-I), while CD4 + T cells activation is driven by MHC II molecules (MHC-II) [78, 79]. Professional antigen-presenting cells (APCs), including dendritic cells (DCs), monocytes, and B cells, internalize and process antigens, producing immunogenic peptides that enable antigen presentation to T lymphocytes. Antigen-specific T cell stimulation is initiated by direct contact of the T cell receptor (TCR) with the immunogenic peptide-bound MHC complexes (pMHC) on antigen presenting cells (APCs) [121]. Some TSPANs have relevant roles during immune responses, including antigen presentation and cell migration.

CD9, CD82, CD37, CD151, CD63, and TSPAN5 have been revealed to interact with MHC complex in APCs. CD9 is reported to associated with MHC II molecules in dendritic cells (DCs) and B cells, which might facilitate the formation of MHC II multimers [122]. Deletion of CD9 in mice enhanced macrophage infiltration and TNF-α production in the lung after administration of Lipopolysaccharide [123]. CD9 knockout bone marrow-derived DCs (BMDCs) induces lower levels of T cell activation than wild-type DCs. CD9 causes MHC-II retention on cell surface by facilitating MHC II trafficking and reducing MHC II endocytosis and recycling [84]. CD82 is upregulated upon activation of BMDCs and monocyte-derived DCs, supporting MHC class II maturation and stable interactions between T cells and splenic DCs or BMDCs through inhibiting RhoA activation [124]. On contradictory, CD37, CD151 and CD63 exhibit inhibitory effect on T cell activation. DCs lacking either CD37 or CD151 expression were hyper-stimulatory to T cells. CD151 inhibits co-stimulation of T cells whereas CD37 dampens peptide/MHC presentation [125, 126]. In addition, knockdown of CD63 in B lymphoblastoid cells consistently activated the CD4 + T-cells via enhancing exosome production [127]. However, it should be noted that CD63 influences neither the amount nor dimerization of MHC II in these cells [87]. CD53, CD81, and CD82 have been revealed to bind with MHC class II molecules in B cell lymphoma cells, but the effect needs further study [81]. Less is known about the association between TSPAN proteins and MHC I complex. One latest study demonstrated that TSPAN5 associates with MHC I molecules to induce more intense MHC I clusters for CD8 + T cell activation. This interaction starts in the endoplasmic reticulum and is maintained on the cell surface [88].

Moreover, CD37, CD81, CD82, CD53 and TSPAN33 regulate the adhesion or migration of APCs. CD37 ablation impairs chemo-tactic migration and in vivo priming of adoptively transferred naive T cells of DCs via activating Rac-1 [128, 129]. CD81 is required for the lamellipodia formation of DCs during migration [130]. CD81 increases adhesion strengthening in monocytes and primary murine B cells, thus facilitating both leukocyte rolling and arrest on VCAM-1 under shear flow as well as adhesion to fibronectin during short stationary contacts [131]. CD82 is upregulated upon activation of BMDCs and monocyte-derived DCs, and restrains migration of BMDCs [129]. Accordingly, CD82 restrains the migration of neutrophils and macrophages into tissues [132]. CD53 could enhance the degranulation of rat NK cells in response to tumor cells, and reduce the IFN-γ response, while decrease homotypic adhesion by activating the β2 integrin LFA-1 [133]. CD53 could also impede the adhesion of both B and T cells [134]. TSPAN33 is reported to promote protrusion formation and invasion in B cells, meanwhile reducing cell adhesion [135]. The effect of TSPANs on T cells remains poorly known. One recent study reported that CD53 could stabilize CD45 on T cell membrane and is required for optimal phosphatase activity and subsequent activation [136].

Although emerging evidence demonstrated that TSPAN proteins are important regulator of immune cells, there lacks direct evidence showing the function of TSPAN proteins in cancer immunology. Further work needs to be done to explore the potential functions. Interestingly, Daniel and colleagues identified that TSPAN8 can be used as a specific target candidate for chimeric antigen receptor T cells (CAR-T) against pancreatic cancer among 371 antigens. CAR-T cells specific for TSPAN8 can significantly decrease the tumor burden in a subcutaneous xenograft model [77].

## Discussion and conclusion

The TSPANs are a family of 33 four-transmembrane proteins in Homo sapiens. TSPANs are mainly expressed on the surface of most nucleated cells and play important roles in cell proliferation, differentiation, adhesion, migration, and cell–cell crosstalk. Recent studies have revealed that TSPANs are indispensable for cancer initiation and progression. TSPANs affect different biological processes mainly via interacting with different partner molecules to form TEMs, including integrins, EGFR, mtTGF-β, EWI2, ASCT2, LC3, PTCH1, P120 and others. We herein summarized the recent studies revealing the versatile role of TSPAN family members in cancer cell invasion, metastasis, proliferation, stemness maintenance, drug resistance, and EV biogenesis. However, other proteins with four transmembrane domains are not included in the TSPAN family, such as TM4SF5 [137].

Most TSPAN proteins are reported to promote cancer invasion and metastasis, such as TSPAN1, TSPAN7, TSPAN8, TSPAN12, TSPAN15, CD151, CD81, CD9, TSPAN31, TSPAN13 and TSPAN9, while CD82, CD63 and TSPAN6 can inhibit cancer invasion or metastasis. The function of TSPAN7 is context-dependent. On the one hand, TSPAN7 can promote the invasion and metastasis of myeloma, non-small lung cancer, and osteosarcoma [72–74]. Conversely, TSPAN7 is downregulated in bladder cancer tissues and inhibits cell proliferation, invasion and in vivo tumor growth [75]. TSPAN15 could promote the proliferation of hepatocellular carcinoma cells [17], but not esophagus carcinoma cells [55]. There are also some discrepancies in the function of specific TSPAN members in cancer cell proliferation and invasion. TSPAN31 and TSPAN7 can promote migration of hepatocellular carcinoma and myeloma cells respectively, but do not affect cell proliferation [30, 66]. More interestingly, TSPAN12 could inhibit the growth of breast cancer cells, but facilitate metastasis [44]. These results showed the complex roles of TSPAN members in cancer metastasis.

CSCs are a distinct population of cancer cells with the capabilities of self-renewal, drug-resistance, metastasis and tumorigenicity. TSPAN1, TSPAN8, TSPAN3, CD9, CD82 have been shown to enhance the stemness of cancer cells. CD63 and CD81 could contribute to the maintenance of hematopoietic stem cells and intestinal stem cells, respectively. Nevertheless, their role in CSCs remains to be explored. TSPAN1, TSPAN3, TSPAN12, CD9, CD81, TSPAN31 and CD82 can mediate chemoresistance in multi cancer types. Meanwhile, CD81 could enhance glioblastoma survival after radiation treatment [100]. We then summarize recent studies showing the essential roles of TSPAN family members in EV-driven cell–cell communication. TSPAN proteins are abundantly enriched in exosomes and can regulate the biogenesis of exosomes, the uptake of exosomes by target cells, and the cargo within exosomes. TSPAN8 has been reported to promote exosome production and the attachment to target cells [103], while TSPAN6 suppresses exosome production and regulate the contents in EVs [115]. TSPANs can regulate the formation of migrasomes, which are smaller vesicles released during cell migration [138]. Currently, the migrasomes exhibit three modes of action: release of signaling molecules through rupturing or leaking, carriers of damaged mitochondria, and lateral transfer of mRNA or proteins [139]. It has been reported that 14 out of the 33 known TSPANs could enhance migrasome formation, but only TSPAN4 has been comprehensively studies. The function of TSPANs on migrasome formation and uptake needs further study.

The immune system is critical in immunosurveillance against cancer. T cells are the focus of tumor immunology as they can efficiently kill cancer cells. The activation of T cells relies on the antigen presentation process [119]. CD9, CD82 have been revealed to interact with MHC II complex in APCs, which might facilitate the formation of MHC II multimers and subsequent CD4 + T cell activation [122]. One latest study demonstrated that TSPAN5 could promote the formation of intense MHC I clusters for CD8 + T cell activation [24]. On the contrary, CD37, CD151, CD63 on APCs exhibit suppressors of MHC presentation [125]. In addition, several TSPAN members, including CD37, CD81 and CD82, are required for the migration of APCs [129]. It should be noted that TSPANs can also regulate attachment, entry, and internalization of viruses, including SARS-CoV-2 [140–142], implying that TSPANs might impact the development of virus-related cancers. Although emerging evidence showed that TSPAN proteins are important for the proper function of APCs, the function of these molecules in cancer immunology is yet to be elucidated. Further, TSPAN proteins may also affect the function of other stroma cells, as TSPAN12 in fibroblasts promotes cancer cell proliferation and invasion through direct cancer-to-stromal cell contact with unknown mechanism [122].

Increasing studies have revealed the regulatory mechanism of TSPAN expression in cancer. The regulation of TSPAN expression can be divided into transcriptional regulation, post-transcriptional regulation, and post-translational regulation. (1) Several transcription factor, like SOX9 and β-cantenin, and promoter demethylase LSD1 have been reported to regulate TSPAN8 transcription. SOX9 could directly enhance the transcription of TSPAN8 expression in response to EGF stimulation [42], while β-cantenin could trigger the direct transcriptional activation of TSPAN8 in melanoma cells [43]. Lysine Specific Demethylase 1 (LSD1) could up-regulate TSPAN8 expression by reducing H3K9me2 occupancy on the TSPAN8 promoter in colorectal cancer cells [41]. (2) The post-transcriptional regulation of TSPANs expression mainly involves microRNAs (miRNAs) and RNA binding proteins. miR-518f-5p could inhibit the expression of CD9 in breast cancer cells [60]. miR-573 and miR-454 could suppress the expression of TSPAN1 in gastric cancer and pancreatic cancer cells, respectively [35, 79]. Meanwhile, TSPAN12 can be the target of miR-495 and miR-196b-5p in lung cancer cells [76, 77]. miR-339-5p has been reported to inhibit TSPAN15 expression in esophagus cancer cells [14], while miR-193a-3p targets TSPAN3 in acute myeloid leukemia [101]. Moreover, miR-135b could depress TSPAN31 expression in hepatocellular carcinoma cells [65]. Finally, RNA binding protein Musashi 2 has been reported to bind with the TSPAN3 mRNA and increase its expression [91]. (3) The post-translational regulation of TSPAN proteins includes glycosylation, palmitoylation and phosphorylation. Glycosylation of TSPAN proteins seems to augment their function. Glycosylation of CD63 in breast cancer cells by RPN2 could increase the cell membrane localization of CD63 [13]. Glycosylation of TSPAN-1 at four distinct sites promotes its correct folding and transition through the endoplasmic reticulum in ovarian cancer cells [21]. Glycosylation of CD82 by the glycosyltransferase MGAT3 is pivotal to disrupt integrin α5β1-mediated cellular adhesion and cytoskeleton rearrangements [22]. Palmitoylation and phosphorylation of TSPAN8 have been reported to facilitate its nucleus translocation, which enhances the chromatin occupancy of STAT3 transcription factor [47].

In conclusion, emerging data has elucidated the critical role of TSPANs in cancer development. Although TSPANs have no natural ligands, they interact with other proteins to elicit their function on cancer cells, ranging from proliferation, apoptosis, migration, invasion, chemoresistance, stemness, to exosome biogenesis. Future work can be done in the following aspects: (1) Elucidate the function of other TSPAN members in carcinogenesis; (2) Demonstrate the upstream and downstream molecular mechanism of TSPANs; (3)Validate the therapeutic efficiency of TSPAN-based strategies, including developing specific antibodies, gene therapy, specific CAR-T cells and others; (4) Reveal the role of TSPANs in the uptake of exosomes; (5) Explore the role of TSPANs in cancer immunology or other stroma cells in the TME; (6) Resolve the structure of TSPAN proteins.

## Data Availability

Not applicable.

## Abbreviations

TSPAN: Tetraspanin
TEM: Tetraspanin-enriched microdomains
EV: Extracellular vesicle
TM: Transmembrane domain
ECL: Extracellular loop
ICL: Intracellular loop
mtTGF-β: membrane type TGF-β
ASCT2: Sodium-Dependent Neutral Amino Acid Transporter Type 2
EFGR: Epidermal growth factor receptor
FATP1: Fatty acid transporter protein 1
MDR1: Multidrug resistance protein 1
SOCSS3: Suppressor of cytokine signaling 3
ATXN3: Ataxin 3
ADAM10: ADAM metallopeptidase domain 10
BTRC: Beta-transducin repeat containing E3 ubiquitin protein ligase
EWI2: Glu-Trp-Ile EWI motif-containing protein 2
NF-κB: Nuclear factor kappa B subunit 1
IκBα: NF-kappa-B inhibitor alpha
EMT: Epithelial-mesenchymal transition
FAK: Focal adhesion kinase 1
Src: SRC Proto-oncogene
PTEN: Phosphatase and tensin homolog
LC3: Microtubule associated protein 1 light chain 3 alpha
SPTLC1: Serine palmitoyltransferase long chain base subunit 1
CDK4: Cyclin dependent kinase 4
MDCK: Madin-Darby canine kidney cells
CSC: Cancer stem cell
ALDHA1: Aldehyde dehydrogenase 1 family member A1
IL-10: Interleukin 10
BTK: Bruton tyrosine kinase
Rad51: Recombination protein A
EE: Early endosomes
MVB: Multivesicular bodies
MHC-I: MHC class I molecules
MHC-II: MHC class II molecules
pMHC: Peptide-bound MHC complexes
APCs: Antigen presenting cells
DCs: Dendritic cells
SOX9: SRY-box transcription factor 9
LSD1: Lysine specific demethylase 1
BC: Breast cancer
GC: Gastric cancer
PDAC: Pancreatic ductal carcinoma
OVC: Ovarian carcinoma
TNBC: Triple-negative carcinoma
ESCC: Esophagus squamous cell carcinoma
NSCLC: Non-small cell lung cancer
SCLC: Small cell lung cancer
AML: Acute myeloid leukemia
BCL: B cell lymphoma
TCL: T cell leukemia
HNSCC: Head and neck squamous cell carcinoma
CCA: Cholangiocarcinoma
PC: Prostate cancer
FA: Fatty acid
